# What do health workers in Timor-Leste want, know and do? Findings from a national health labour market survey

**DOI:** 10.1186/s12960-016-0164-1

**Published:** 2016-11-18

**Authors:** Xiaohui Hou, Sophie Witter, Rashid U. Zaman, Kay Engelhardt, Firdaus Hafidz, Fernanda Julia, Christophe Lemiere, Eileen B. Sullivan, Estanislau Saldanha, Toomas Palu, Tomas Lievens

**Affiliations:** 1 World Bank, Washington, DC United States of America; 2 Oxford Policy Management, Oxford, United Kingdom; 3 Queen Margaret University, Edinburgh, United Kingdom; 4 Universitas Gadjah Mada, Yogyakarta, Indonesia; 5 Dili Institute of Technology, Dili, Timor-Leste

**Keywords:** Health workers, Doctors, Nurses, Midwives, Motivation, Competence, Performance, Timor-Leste

## Abstract

**Background:**

The objectives of this study were to understand the labour market dynamics among health workers, including their preferences and concerns, and to assess the skills, competence and performance (i.e. the ‘know–do gap’) of doctors working in Timor-Leste.

**Methods:**

This cross-sectional survey was implemented in all 13 districts of Timor-Leste in 2014. We surveyed 443 health workers, including 175 doctors, 150 nurses and 118 midwives (about 20% of the health workers in the country). We also observed 632 clinical consultations with doctors, including 442 direct clinical observations, and tested 190 vignettes.

**Results:**

The study highlights some positive findings, including the gender balance of health workers overall, the concentration of doctors in rural areas, the high overall reported satisfaction of staff with their work and high motivation, the positive intention to stay in the public sector, the feeling of being well prepared by training for work, the relatively frequent and satisfactory supervisions, and the good attitudes towards patients as identified in observations and vignettes. However, some areas require more investigations and investments. The overall clinical performance of the doctors was very good in terms of attitude and moderate in regard to history taking, health education and treatment. However, the average physical examination performance score was low. Doctors performed better with simulated cases than the real cases in general, which means they have better knowledge and skills than they actually demonstrated. The factors that were significantly associated with the clinical performance of doctors were location of the health facility (urban doctors were better) and consultation time (cases with more consultation time were better). Regression analysis suggests that lack of knowledge was significantly associated with lack of performance, while lack of motivation and equipment were not significant.

**Conclusions:**

The survey provides essential information for workforce planning and for developing training policies and terms and conditions that will attract and retain health workers in rural service. Improving the work environment and performance of doctors working in rural health facilities and ensuring compliance with clinical protocols are two priority areas needed to improve the performance of doctors in Timor-Leste.

## Background

Timor-Leste represents a unique case to study human resources for health as it is one of the few countries in the world which has rapidly mobilised resources and has addressed the shortage of primary care doctors. During the war preceding independence in 2002, more than 70% of the country’s health facilities was destroyed or seriously damaged and only approximately 20 doctors remained in the country for nearly 900,000 people [1].

The Government of Timor-Leste had a strong political commitment to combat the shortage of doctors. In 2003, the government signed an agreement with the Cuban Government to train and deploy medical doctors throughout the country. Since 2010, the newly trained doctors have been deployed annually throughout Timor-Leste upon successful completion of their training programme. The total health workforce has thus increased significantly, with the doctor-to-1000 population ratio at 0.8 per 1000 population in 2014, which is comparable to other countries in the region [2, 3]. At the same time, the nurse-to-1000 population ratio remained 0.9 per 1000 population, lower than some countries in the region [2].

Medical students were recruited from the country and were trained either in Cuba or Timor-Leste, by the Cuban Medical Brigade (CMB) since 2004. Until 2007, medical students were trained simultaneously in Cuba and Timor-Leste. Students enrolled in or after 2008 are trained exclusively in Timor-Leste by CMB in collaboration with the University of Timor-Leste (UNTL). The programme is a 6-year Bachelor of Medicine course consisting of 5 years of medical schooling and 1 year of internship in the health facilities across the countries. The important characteristic of the Cuban medical training programme is a community-based primary health care approach. After graduation, the medical doctors sign an agreement with the government to work in the public health sector for at least 6 years.

Timor-Leste has a three-tiered government health system, which includes a national hospital at the tertiary level, district hospitals at the secondary level and community health centres (CHCs), and health posts (HPs) at the primary level. Outreach centres known as Servisu Integrado du Saude Comunidade (SISCa) also operate at the primary level [4]. According to the information provided by the Ministry of Health (MoH), there were 5 district hospitals, 66 CHCs, 205 functioning HPs and 442 SISCas in-country at the time of the survey. However, there are reports that not all of the SISCas were fully functioning [5]. The private health system is not very well developed in Timor-Leste, particularly in rural areas. In 2011, there were 26 private health facilities in Timor-Leste, mostly in urban areas [6]. Government health workers are officially prohibited from private practice in Timor-Leste.

The doctors trained were deployed to various levels of public health facilities. Most of them were supposed to return to the health posts in the communities from which they came. With this influx of large number of doctors, the critical shortage of health workers has lessened and the overall presence of doctors in rural areas has hugely improved. However, the presence of doctors on the ground does not automatically translate to improved service delivery. More complicated issues are emerging in recent years that are related to human resource management, retention in rural facilities and the quantity and quality of services provided by health workers.

Similar to most developing countries, Timor-Leste faces challenges of retention of health workers but it also has some unique features. Various push and pull factors influence health workers’ choices to decide to stay or not stay in rural health facilities. According to the strategic development plan of the Government of Timor-Leste, all health posts should have at least one doctor, two nurses and two midwives by 2020 [7]. Most of the Cuban-trained medical students were enrolled from rural areas and therefore have some incentive to go back to their areas of origin. However, because of the poor functionality of the rural health facilities, doctors may be unwilling to work in those facilities. There are also concerns regarding a lack of supervision and unclear career development paths for those working in rural areas [8].

There is lack of data on the utilisation of the services and the productivity of health workers posted in rural areas. A recent critical appraisal estimated that the patient load at the primary health care level in Timor-Leste was on average less than three patients per day [9]. However, this finding was based on modelling, not primary data. In general, the supplies, management, supervision and opportunities to learn in rural health facilities in Timor-Leste may require improvement and health workers deployed in these facilities may not be sufficiently motivated. An article published on the tuberculosis (TB) services in Timor-Leste indicated that there was a lack of ownership by government health workers [10].

There are also some concerns about the quality of care of the Cuban-trained doctors [6]. A study was conducted on the use of medicine and adherence to the clinical guidelines by non-physician heath workers (e.g. nurses and midwives) in Timor-Leste and it showed favourable results [11]. However, there is no evidence on the clinical performance of doctors that could further guide the on-the-job training of the current physician workforce.

All the above information indicate that there is a general lack of evidence in various aspects of the health labour market in Timor-Leste. This lack of information is mainly caused by the absence of an integrated information system and lack of properly designed research [1]. A need for a properly designed nationally representative survey of health workers in Timor-Leste was therefore identified, not only to gather evidence on some of the complex issues like motivation, preference and rural retention but also to systematically assess the quality of care provided by the doctors working in the country to guide policy development.

The objectives of this study were to understand the labour market dynamics among health workers, including their preferences and concerns, especially regarding their revenues and rural jobs, and to assess the skills, competence and performance (i.e. the ‘know–do gap’) of doctors working in Timor-Leste. This is the first study of its kind in Timor-Leste and provides important insights for health market analysis and policy-making. It also adds to the small but growing international published literature on health worker motivation, retention and performance in low- and middle-income countries, including whether there is a ‘know–do gap’ for health practitioners and, if so, what drives it and what can overcome it [12].

## Methods

### Design

The overall study design was a cross-sectional survey including four components: a health facility survey, a health worker survey, a discrete choice experiment (DCE) and a quality-of-care component (including clinical observations and vignettes). A qualitative study was carried out prior to the main study to inform the DCE design. This article presents findings from two components of the study: the health worker survey and the quality-of-care component. The DCE results are published in a separate paper [13].

### Tools

In the *health worker survey*, we gathered data using a structured questionnaire on health workers’ (doctors, nurses and midwives) demographic characteristics and on various aspects of their profession. The field team members administered a structured questionnaire to the sampled health workers within the health facilities for this component. The survey questionnaire included modules on job history, preferences and views on the profession, current job, training, supervision and absenteeism.

The *quality-of-care component* only involved medical doctors. Direct clinical observations (DCOs) were used to evaluate the doctor’s performance in a real clinical setting, alongside vignettes to measure the doctor’s clinical knowledge and skill in an ideal outpatient setting using a standard simulated ‘patient’. We recruited three medical researchers from the University of Gadjah Mada (Indonesia) to administer the DCO and vignette tools. DCOs and vignettes were used for general medical doctors only.

In the DCOs, the medical researchers silently observed the entire clinical consultation of the patients by sampled doctors, using a structured tool to record the attitude of doctors, history taking, physical examination, diagnosis, treatment and health advice. At the end of the consultation period, for the same sampled doctors, the medical researchers observed three simulated cases (fever, cough and diarrhoea) where the enumerators presented themselves as patients. The doctors recorded observations in a structured tool based on the attitude, history taking, physical examination, diagnosis, treatment and health advice of the simulated consultation.

### Sampling

The sampling frame was obtained from the human resource department of the Ministry of Health which was comprised of 2247 health workers including 612 doctors, 1095 nurses and 540 midwives. A nationally representative sample of 443 (20% of total health workforce) health workers was drawn from six strata: two kinds of facilities (urban and rural) and three kinds of health workers (doctors, midwives, nurses). The health workers were sampled using proportionate-to-size (PPS) calculations from 69 health facilities, including the national hospital, all 5 referral hospitals, 33 community health centres and 30 health posts. These health facilities were located in all 13 districts of Timor-Leste.

We sampled the health workers from the list that we had received from the Ministry of Health. However, we observed during the pretesting that the list was often outdated and internal transfers within the districts are often not notified to the central level. Some health workers were also on leave or on training at the time of the survey. In order to avoid high attrition, we used a systematic replacement procedure where the health workers were replaced from the same strata when the originally sampled health workers were not present.

Additional subsamples were drawn for DCOs and vignettes. One doctor was randomly sampled (from the list of sampled doctors) from each facility for this component. The specialist doctors were excluded for this component as their clinical skills are not comparable with general doctors. All the patients who were consulted by the sampled doctors during the day of the survey were enrolled for DCO. However, if the anticipated number of patient load was high, then 10 patients were sampled using systematic random sampling during the fieldwork. For the vignettes, each sampled doctor was presented with three simulated cases (fever, cough and diarrhoea).

### Data collection and analysis

Three field teams collected the quantitative survey data between July and August 2014. Each field team was composed of a supervisor, a medical researcher and two enumerators.

Data were double entered using a customised data-entry programme. Since the samples were drawn from various strata, we used weights to correctly represent the survey population. We used SPSS and Stata to generate descriptive statistics and to perform regressions to see the associations between various predictor and outcome variables. In the DCOs and vignettes, in order to create a single score for each observation, we constructed the total score from each section: attitude, history taking, physical examination, therapy appropriateness and health education.

To find the causes of low-quality scores obtained from the inverted DCO score, we categorised the causes of low quality by three factors: (i) doctors lack knowledge (as can be checked with the inverse of mean vignettes score for each doctor), (ii) doctors are unable to perform because supplies/equipment are missing (as measured by the composite score of equipment and supplies in the health facility survey), and (iii) doctors are making limited effort, due to challenges or lack of motivation (as measured by the score generated by motivation-related questions in the health worker survey). Each variable had equal weights to generate the composite scores, and regression analysis was carried out to see the association.

## Results

We interviewed 443 health workers from 69 health facilities from all 13 districts of Timor-Leste (Table 1). Among the participants, 175 (40%) were doctors, 150 (34%) nurses and 118 (27%) midwives. Out of the 443 sampled health workers, 178 (40%) were male and 265 (60%) were female. Of them, 242 (55%) were working in urban health facilities and 201 (45%) in rural facilities. Of the 175 doctors, 108 (62%) were trained in Cuba, 52 (30%) in Timor-Leste, 12 (7%) in Indonesia and the remaining 3 in other countries. Seventy-one percent of the nurses and midwives were trained in Timor-Leste and 29% in Indonesia.

**Table 1:** Characteristics of the participants

	Total	Doctors	Nurses	Midwives
Number of respondents	443	175	150	118
Sex				
Male	178	74	104	0
Female	265	101	46	117
Locality				
Urban	242	106	71	65
Rural	201	69	79	53
Training location				
Timor-Leste	242	52	107	83
Cuba	110	108	2	0
Indonesia	85	12	39	34
Others	6	3	2	1

Overall, 20% of the doctors were working in HPs, 47% in CHCs, 14% in district/regional hospitals and 19% in the national hospital. The nurses and midwives were mostly working in the CHCs (nurses 60%, midwives 54%) and district and regional hospitals (14–17%). Only a minority of the nurses were working in the HPs (8%, nurses 16%).

Since there were only a few doctors in-country in Timor-Leste at the time of independence, almost the entire cohort of doctors was newly trained; 95% of them had less than 5 years of experience in the sector. However, 45% of the nurses and midwives had 15 or more years of experience.

For the quality-of-care survey, 635 cases were observed, including 442 DCO and 193 vignette cases. Among all, 255 (40%) were in urban areas and 379 (60%) were in rural areas. In terms of the types of health facility, we observed 128 (20%) case patients at hospitals, 305 (48%) cases at CHCs and 199 (31%) cases at HPs.

### Motivation to join, career paths and long-term preferences

The vast majority of all staff types (doctors 98%, nurses 98%, midwives 97%) selected health care as a career in order to help people. Generally, health workers indicated high levels of satisfaction: only 4% of the respondents indicated that they were ‘unsatisfied’ or ‘very unsatisfied’ with their work. Motivation is also shown by the fact that, regardless of staff type, 8 in 10 health workers mentioned that they would stay in the facility until the last patient is treated — even if they do not receive additional money for this.

The survey shows that health workers got opportunities to advance to higher types of facility with more experience. Thirty-two percent of the respondents who work in HPs had more than 10 years of experience. In higher-level facilities (health centres, referral hospitals or the national hospital), the numbers of staff with that level of experience were much higher (61, 59 and 52%, respectively). It therefore appears that staff with comparatively less experience start their careers in HPs.

In the long run, the majority of respondents would like to continue to work in the government/public sector, including 99% of the doctors (Table 2). This was also the case for the broad majority of nurses and midwives, although 6% of nurses would prefer to move to the private sector. This was especially true of those working in HPs. HPs were the least attractive stations for health workers, with only 6% of doctors stating a HP as their long-term preferred facility, although 32% were content to work in CHCs. Only 22% see themselves working in rural areas in the long term. However, 88% acknowledged that it is up to the Ministry of Health (MoH) to determine where their next assignment will be. The majority of respondents (97%) were not looking for another job in the short run (doctors 100%, nurses 96%, midwives 100%). Only very few (4%) had ambitions to leave their clinical position and move into management (1% of doctors and 6% of nurses).

**Table 2:** Long-term goals, by occupational group

	Total (%)	Doctors (%)	Nurses (%)	Midwives (%)
Public sector	95	99	93	96
Private sector	4	1	6	2
NGO	0	0	0	1
Others	1	0	1	0

### Salary and financial benefits

All medical staff receive their money through a direct deposit to their bank account, and only very few (2%) have experienced any delays in receiving their money (nurses and doctors 1% each, midwives 4%). Doctors had an average monthly income of US$ 622, nurses US$ 453 and midwives US$ 479. Wage differentials within each cadre were relatively small, and income did not vary much by the years of experience, particularly for the doctors. On average, a doctor with more than 10 years of experience earned just US$ 50 more than a newly joined doctor. With the exception of nurses, incomes were slightly higher for those who worked in urban areas, reflecting the fact that more senior health workers worked in urban facilities.

More non-financial benefits (such as housing and motorbikes) were reported by doctors and at lower-level facilities (Table 3). However, only half of them reported that they get either sufficient fuel or allowances to buy fuel for the motorbike, and more than half of all respondents also stated that these benefits were delayed.

**Table 3:** Kinds of benefit received, by occupational group

	Total (%)	Doctors (%)	Nurses (%)	Midwives (%)	Hospitals (%)	CHCs (%)	HPs (%)
Housing	17	36	6	20	11	15	38
Housing benefit	2	4	1	1	4	1	0
Motorbike	27	53	22	8	3	38	40

More than half of the health staff interviewed believed their salary to be too low. However, they did not see other labour market opportunities they would prefer. Very few report private practice (but this may be under-reported as private practice is not legally permitted in Timor-Leste).

### Workload

Although most respondents worked 5 days a week in health posts, community health centres and district/regional hospitals (63, 66 and 54 percent respectively), 22% to 36% worked 6 and sometimes 7 days a week. Looking at the number of working hours per day, it becomes apparent that working in a rural area usually means an 8-h working day (97%). In urban areas, on the other hand, the pattern is not so clear cut; 12% of respondents work more than 8 h and another 8% less than that.

We counted the number of patients for the sampled doctors who participated in the DCOs, and the mean number of patients per day was 10.2, with a standard deviation (SD) of 7.5. In urban facilities, the mean patient load was 11.5 (SD 6.9), compared to 9.6 (SD 7.8) in rural facilities. The number of patients also varied by level of the facility, with higher-tier facilities having more patients. Forty-five percent of respondents agreed with the statement that they have ‘too much work to do’. However, we found no clear correlation between workload and feelings of overload.

### Training

Almost all respondents believed that their training prepared them well to diagnose and treat clinical cases in Timor-Leste (doctors 99%, nurses 97%, midwives 95%). When asked how many short-term training sessions (below 30 days) they had attended in the last 3 years, roughly half of the nurses and midwives report doing three or more, while one quarter did not attend any such training in this timeframe. At the same time, roughly one third (35%) of the doctors attended three or more training sessions and around the same proportion (37%) attended none. The doctors located in urban areas were found to benefit more: roughly half (51%) of them had attended three or more training sessions in the last year, as opposed to 26% in rural areas. Despite all the training opportunities, roughly half (52%) of the respondents stated that ‘there is not enough opportunity to learn current medical knowledge’.

**Fig. 1:**
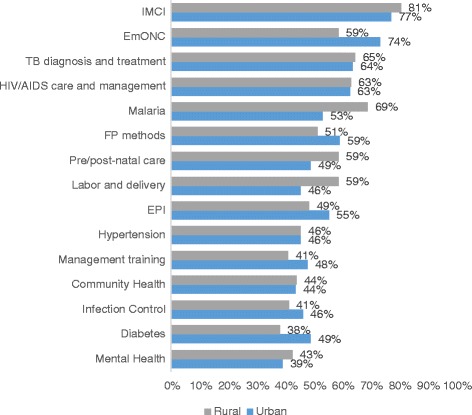
Area of training needs reported by the doctors

Although a large number of doctors were in rural areas, it is interesting to see that only 19% underwent training on community health. Around 75% of doctors indicated that they require training on Integrated Management of Childhood Illness (IMCI) and 64% on Emergency Obstetric and Newborn Care (EmONC). Even though 55% of doctors stated that they received training on tuberculosis (TB) in the last year, roughly two out of three think they require additional training on that subject. Community health, diabetes and mental health were the least desired training topics, yet nonetheless, more than 40% of the doctors requested them. There were only small differences in training requests between doctors in rural and urban areas (Fig. 1).

Older or highly satisfied doctors tended to be less interested in any kind of training, while females were keener than males to have the opportunities for visits from specialists. Workers in health posts and community health centres were more interested in specialisation. Highly satisfied doctors had a lower demand for all kinds of training. Older nurses and midwives, as well as those with more medical experience, tended to be more interested in any kind of training. Rural facility health workers were significantly more willing to finish their bachelor’s degrees.

### Supervision

Eighty-five percent of respondents indicated that they have a supervisor who is responsible for providing feedback on their performance The number of doctors with a supervisor is lowest among the three cadres (doctors 81%, nurses 87%, midwives 85%). Roughly 8 out of 10 respondents (83%) reported that they had meetings with their supervisor at least every 3 months, with the majority of staff—except for midwives—having monthly meetings.

**Fig. 2:**
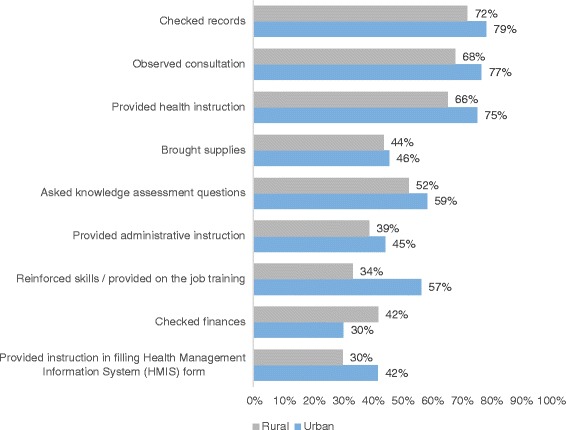
Supervisor activities by locality

Supervisors performed a wide range of duties during the supervision visits. The top of the list of tasks for supervision was an administrative task: checking records (Fig. 2). It appears that the nature of supervisory visits is by and large the same in rural and urban areas, although ‘checking finances’ seems to be more prevalent in rural areas whereas ‘on-the-job training’ happens more in urban settings. Seventy-five percent of all respondents (doctors 69%, nurses 79%, midwives 72%) stated that they had felt the need to discuss difficulties with their supervisor within the last year; most of them (95%) actually discussed it with him or her and 66% noticed improvements afterwards. Given that, it is not surprising that the majority of respondents (77%) were ‘(very) satisfied’ with the last supervisory meeting and their motivational effect is also high: 86% indicate that they ‘always’ feel motivated afterwards. Overall, it appears that these sentiments exist across all categories, with few significant differences between the type of staff, locality or the facility type the respondent works in.

### Absence from work

Eight percent of respondents reported being absent from work in the 30 days before the interviews because they were sick. This was the same across all types of health workers. However, more than 33% of doctors stated that they had attended training and were therefore absent from their work; this proportion was lower for nurses and midwives (with 17 and 28% indicating they had attended training, respectively).

Across all staff types, roughly 5% of them were absent in that timeframe because of personal reasons. In the event of an absence, roughly 14% of all staff types stated that they were called by the facility head. In 5% of the cases, the supervisor discussed the absence with staff and 2% of nurses and midwives indicated that money was deducted from the salary as a sanction — this did not happen for the doctors.

### Perceived challenges

**Fig. 3:**
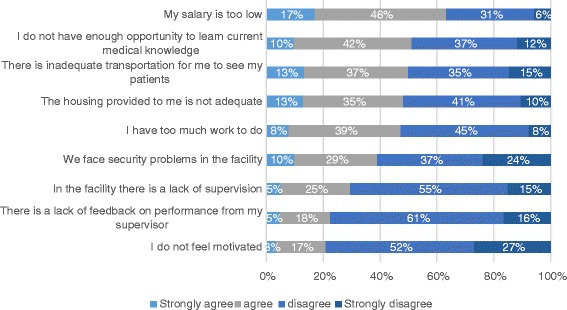
Problems in the current job

Low salaries were raised as a problem by all health workers faced in the job (with 63% agreeing or strongly agreeing with the statement ‘my salary is too low’), although the majority of the doctors are satisfied with their salaries (doctors 58%, nurses 25%, midwives 38%). This was followed by inadequate opportunities to learn (52%), lack of transport to see patients (50%), inadequate housing (48%), too much work (47%), security problems (39%), lack of supervision (30%), lack of feedback on performance (23%) and lack of motivation (20%) (Fig. 3). Lack of motivation was correlated most closely with perceptions of being overloaded (0.27). Nurses and midwives were more likely to report inadequate transport, while nurses were most critical of salary levels.

### Knowledge and practice scores

The overall clinical performance as measured by the combined score of DCO and vignettes of general practitioners was very good in terms of attitudes (91% score) and moderate at history taking (57%), health education (50%) and treatment accuracy (69%). However, the average physical examination performance score was low at 28%. The overall average performance score was 62%.

When comparing observations with vignettes (to assess knowledge), there were significant differences in history taking, appropriateness of treatment and health education (Table 4). The average scores for history taking and health education were higher in the vignette group, suggesting a ‘know–do’ gap. However, scores on the appropriateness of treatment were much better for DCOs than vignettes, which probably mean that vignette cases were harder than daily practice cases or were not well understood.

**Table 4:** Difference in scores between DCOs and vignettes

	DCO	Vignette	*P* value
Attitude	0.91	0.92	0.69
History taking	0.54	0.65	0.00^a^
Physical examination	0.23	0.37	0.00^a^
Treatment	0.74	0.59	0.00^a^
Health education	0.46	0.60	0.00^a^
Total score	2.85	3.11	0.00^a^

^a^Significant

Both observations and vignettes revealed a low rate of hand washing before and after patient consultations—slightly above 20% in observed cases and below 10% for simulated vignettes.

### Determinants of clinical performance

When the clinical performance was distinguished by the type of health facilities, health posts had a negative correlation with clinical performance. As health posts were found to be comparatively poorly equipped, doctors might face difficulties in performing ideal clinical practice. The location of the practice also had a significant effect on the clinical performance of doctors; doctors working in a rural area were less well performing than their counterparts in urban areas, which again may link to the absence of an enabling work environment.

The clinical performance was also better when consultation periods were longer. The average consultation time per case was 9 min. Doctors spent less time in DCOs (8 min) compared to vignettes (10 min), which was statistically significant (*P* ≤ 0.01). Doing the vignettes after work may have permitted this longer focus on each case.

The knowledge of the medical doctors showed a significant relationship to their performance (34% correlation with non-quality). On the other hand, the medical doctors’ low effort and lack of equipment, drugs and supplies did not show a significant relationship to the non-quality score of performance (Table 5).

**Table 5:** Determinants of doctors’ performance gaps

	Correlation coefficient (*r*)	Significance (*P* value)
Lack of knowledge	0.34	0.00^a^
Lack of motivation	0.07	0.13
Lack of equipment and supplies	0.06	0.19

^a^Significant

## Discussion

The study highlights some features of Timor-Leste which are relatively unusual and relate to the recent history of the country. There are indeed few instances where health workers from low- and middle-income countries (LMICs) demonstrate a high job satisfaction [12]. The young age of the medical cohort, and their concentration in rural areas, is a legacy of its recent independence and policies pursued since, including a reliance on Cuban training and Cuban Brigade doctors. The high levels of reported supervision, its substantial content and satisfaction with supervision are also encouraging. The findings suggest that policies have been successful in creating a motivated workforce in general, perhaps as a result of the training programme ethos [8] or that they are motivated by being back in their rural communities. However, the age of the cohort and the short time in service may also be important explanatory factors, along with the newly independent status of the country, which encourages a public service ethic, and the relative absence of other employment options in the health labour market. For instance, there is widespread evidence in LMICs that physicians start engaging in dual-job holding or move to the private sector only once they get older (when they have gained a reputation through their public sector job and/or have more dependents and thus need to earn higher incomes) [14]. That could explain why these young physicians are happy with their job, for now.

The study has been able to triangulate clinical observations and vignettes for doctors with self-reporting to better understand the determinants of performance as pioneered by Leonard and Masatu [15]. Health workers’ confidence and attitudes received high scores, but improvements can be made in the skills and enabling environment for other aspects, such as physical examinations in particular. Analysis appears to confirm that specific training and better supplies and equipment are the main areas to address and that the component of effort is not significantly responsible for current performance gaps.

However, as consultation time was one of the significant determinants, it can be assumed that the clinical performance of doctors can be improved by ensuring compliance with clinical protocols. The data suggest that doctors’ patient load was not too high (on average, 10 patients per day). Therefore, time constraints should not be a reason for not spending sufficient time on each patient and following the appropriate clinical protocol for the diseases in question.

It is also evident from this and another study that, even though the government has provided housing and transportation facilities, doctors working in rural areas face many difficulties in their daily life [16]. Sometimes they run out of water and electricity both in their houses and clinics, and it is difficult for them to travel due to poor roads. Moreover, supplies of medical equipment, stocks of drugs and also the number of medical professionals in rural areas are limited. These factors may all contribute to doctors’ clinical performance and hence need to be addressed to retain staff over time.

While the frequency of the supervision visits was high, the impact of that in day-to-day clinical practice is not very clear. For example, despite a high level of supervision visits, the hand-washing rate was quite low (results shown under knowledge and practice scores). This indicates a potential lack in the quality of supervision or the supervision visits being overly focused on administrative and managerial aspects. This assumption correlates with the findings of 52% health workers who reported inadequate opportunities to learn and checking records being the most frequent activity of the supervision visits. In this regard, we recommend strengthening the quality of supervision with enhanced focus on clinical supervision.

There are a number of areas of internal contradictions in our findings, which merit more reflection—for example, health workers report positively on initial training and report relatively frequent in-service trainings but also demand more training. It is not clear whether this reflects a demand for more knowledge or whether training has other benefits for staff. For instance, other similar surveys have shown that opportunities to further specialise (possibly to get a private job) are highly valued among physicians or nurses [17]. Overall, however, our findings fit with the wider literature which emphasises that financial incentives, career development and management issues are core factors in motivating health staff and that continuing education has particular significance for young professionals [18].

The study has several limitations. One of them is the lack of demand-side information; our sample only included the health facilities and health workers and did not include any patients’ perspectives. We did not include private health providers in this survey, which is another important limitation. About one quarter of the health services in Timor-Leste are provided by private health care providers, and including them would have increased the comprehensiveness of the survey. However, the primary goal of this survey was to inform policy on government health workers.

The assumption that vignettes capture knowledge and DCOs performance (thus the two sides of the ‘know–do’ relationship) is also open to discussion, as illustrated by some of our results. Some aspects of performance were better in practice than in theory, which technically should not be possible. That shows that these research tools may be capturing a range of features, including the effects of different framing of problems, as well as the different contexts. Although vignettes have been validated in other settings [19], our results suggest they should be interpreted carefully.

## Conclusions

Timor-Leste has made a remarkable difference by rapidly increasing health workers’ presence in the country, particularly in the rural areas. However, the presence of doctors on the ground is only the necessary but not sufficient condition to deliver high-quality services. This study finds significant gaps in some areas of doctors’ performance, particularly in relation to physical examinations. Our findings suggest that low motivation is not a factor but that lack of knowledge and poor facilities contribute to non-performance. This will require the Government of Timor-Leste to strengthen specific aspects of training, supervision, compliance with protocols and functionality of health facilities, especially in rural areas. These findings are likely to have wider applicability to similar low-income contexts. The study also illustrates the potential and the challenges of surveys which attempt to measure multiple possible determinants of health worker performance.
